# Depression and Breast Cancer in Morocco: Prevalence and Associated Factors

**DOI:** 10.1155/2023/3277929

**Published:** 2023-11-27

**Authors:** Khadija Benallel, Rajae El Kilali, Roukaya Benjelloun, Mohamed Kadiri

**Affiliations:** ^1^Psychiatry Department, Mohammed V Military Hospital, Faculty of Medicine and Pharmacy of Rabat, Mohammed V University, Rabat, Morocco; ^2^Ibn Sina University Hospital of Rabat, Faculty of Medicine and Pharmacy of Rabat, Mohammed V University, Rabat, Morocco; ^3^Psychiatry Department, Cheikh Khalifa International University Hospital, Mohammed IV University of Health Sciences, Casablanca, Morocco

## Abstract

**Background:**

Depression is frequently associated with breast cancer. However, its prevalence and impact on patients' quality of life are negligible. Depression is often underdiagnosed and less treated.

**Objectives:**

Our study is aimed at estimating the prevalence of depression in breast cancer patients, describing their sociodemographic and clinical profile, and determining the factors associated with this depression. *Material and Methods*. We carried out a cross-sectional, descriptive, and analytical study, conducted from January to March 2018 at Mohammed V Military Hospital in Rabat. The survey included 100 breast cancer patients. It was conducted using an anonymous questionnaire, the Mini International Neuropsychiatric Interview test (MINI test) to diagnose depression and the Beck Depression Inventory (BDI) to assess its severity.

**Results:**

The major depressive episode was diagnosed in 26% of breast cancer patients. Age under 40, psychiatric history, metastatic breast cancer, refusal of treatment, heavier treatment, and spousal alteration were the factors most associated with higher depression prevalence in these patients. *Discussion and Conclusion*. The high prevalence of depression in breast cancer patients, as well as the influence of personal characteristics and treatment in the occurrence of this ailment, has been confirmed by several authors. It is recommended to improve the psychooncological care of patients with breast cancer, to prevent the occurrence of depression in this vulnerable population.

## 1. Introduction

Breast cancer is the most common cancer in women and a significant public health problem. Its worldwide incidence is estimated at 1.5 million new cases diagnosed each year with a mortality of nearly 570,000 deaths [1]. It is a dual source of physical and psychological suffering, particularly depression. Indeed, depression is the leading cause of morbidity and disability in the world. More than 300 million people suffered from it with an increase of more than 18.4% from 2005 to 2015 [2, 3]. Depressive disorders are two to three times more common in cancer patients than in the general population [4]. However, breast cancer is considered among the most neoplasms associated with a higher prevalence rate of depressive episodes, with a percentage ranging from 1.5 to 46% [5, 6].

The psychological suffering of women with breast cancer has a particular dimension because it is linked on the one hand to the image of cancer and its treatment, which is associated with suffering and death, and on the other hand to the image of breasts as symbols of femininity, maternity, and sexuality. These dual symbolic references will influence the occurrence of depression in this vulnerable population. However, this condition is often underdiagnosed in these patients, partly because feelings of sadness and discouragement are often considered normal and appropriate when faced with the awareness of a cancer diagnosis and partly because many signs are common to both cancer and depression, such as weight loss, fatigue, or sleep disturbances. The main objectives of this study were to estimate the prevalence of depression in a population of breast cancer patients, to describe their sociodemographic and clinical profile, and to determine possible factors influencing the occurrence of this psychological condition.

## 2. Materials and Methods

Our survey was a descriptive cross-sectional study extending from January to March 2018. It took place in two departments, radiotherapy and medical oncology of the Mohammed V Military Hospital in Rabat (HMIM V). It included 100 patients. They all had documented breast cancer at different stages of the disease. They were randomly selected and informed of the purpose of the study. Only patients with free and informed consent were recruited. Included were female subjects, older than 20 years. They all were aware of their cancer disease, followed, and treated. Male patients, women under 20, and those with impaired general conditions were excluded. All participants gave their consent after explaining the principle and the aims of the study.

We developed an anonymous questionnaire on sociodemographic characteristics, breast cancer characteristics (location, stage, and circumstances of discovery) and its therapeutic management, and the psychological, physical, socioeconomic, and family repercussions after discovering breast cancer. The diagnosis of depression was established by using the Mini International Neuropsychiatric Interview test (MINI test), and its severity was assessed by using the Beck Depression Inventory (DBI). It is a 13-item instrument; each item is scored from 0 to 3 for a total score ranging from 0 to 39. The questions were asked in dialectal Arabic, and those of the MINI test [7] and the DBI [8] were reformulated in Arabic. All data were entered into Excel 2016 and analyzed using SPSS 20.0 (Statistical Package for the Social Sciences). The qualitative variables were expressed as headcount (*n*) and percentage (%) in the descriptive part. In the statistical analysis part, we used the chi-2 test to compare qualitative variables. A difference was considered statistically significant when the *p* value was strictly less than 0.05 (*p* < 0.05).

This study's principle and questionnaire were included in a medical thesis that was approved by the scientific committee of theses and registered under M1672018 at the library of the Medicine Faculty of Rabat in Mohammed V University in Morocco [9].

## 3. Results

Our study involved a sample of 100 patients, all with breast cancer. The majority of our population (72%) was between 40 and 60 years of age. 82% were married and 45% of them were illiterate. 82% were housewives. 57% reported a medium socioeconomic level. All patients had medical insurance (full cover for cancer and all other chronic diseases, including psychiatric disorders) and a regular income (although 82% of the patients had no job, they were all wives of active or retired public workers). Regarding the patients' history, the majority (75%) reported no personal medical history. 59% of them had no surgical history, and only 17% of patients had a psychiatric history. In addition, no patient reported a history of psychoactive substance use. As for family history, most patients had neither a family history of similar cancer (83%) nor a family history of depression (88%) (Table 1).

Regarding the characteristics of breast cancer, the majority of patients (94%) had cancer located in one breast only. Half of them had cancer that had spread locally, and 24% had metastatic disease. In addition, 89% of the women reported that they discovered their breast abnormality by self-examination. 28% of the patients benefited from surgery and chemotherapy, 20% benefited from surgery and chemotherapy associated with radiotherapy and 18% received chemotherapy only (Table 1).

Regarding the psychological and behavioural repercussions after the discovery of cancer, several causes of discomfort were reported on different levels. The leading cause of psychological discomfort was feelings of sadness (88%). As for physical discomfort, most patients (73%) attributed it to physical symptoms (pain, asthenia, and sexual problems). On the other hand, on the social, family, and economic level, the cause of discomfort reported by 28% of them was the insouciance of the family.

Finally, regarding the study of depression, according to the MINI test, 26% of breast cancer patients had a characteristic depressive episode at the time of the survey. According to the BDI, among the 26 depressed patients, 42% had mild depression, 50% had moderate to severe depression, and 8% had severe depression. Only 2% of all patients had received psychological care from a psychiatrist.

We analyzed the data collected in our survey by comparing the prevalence of depression in breast cancer patients according to the following factors: sociodemographic characteristics, personal and family history, clinical characteristics of breast cancer, therapeutic management, and psychological and behavioural impact. Age between 20 and 40 years was significantly correlated with the development of depressive disorders (*p* < 0.05). Indeed, the majority of women aged between 40 and 60 years were not depressed, while no patient aged over 60 years presented a depressive disorder. This association between age above 60 and the absence of depression was also statistically significant (*p* < 0.05). Furthermore, there was no significant correlation between the occurrence of depression in our sample and other characteristics, namely, marital status, having children or not, area of living, education level, professional status, and socioeconomic level. Regarding the personal and family history of the patients, only the personal psychiatric history proved to be a risk factor for the occurrence of depressive disorders, and this was statistically significant (*p* < 0.05) (Table 2).

The circumstances of cancer discovery and location did not predict depression. However, the stage was found to be directly correlated with the risk of depression with a high level of significance (*p* < 0.05). Indeed, patients with metastatic breast cancer had more depressive disorders than those with locally diffused or locoregional cancer.

At the time of the diagnosis, several reactions or feelings were expressed by the patients, but only side ration and refusing treatment were significantly related to depression (*p* < 0.05). The feeling of shock was correlated with fewer depressive disorders, in contrast to refusal of treatment, which was related to a higher frequency of these disorders (Table 2).

The study of the parameters related to therapeutic management showed that they were all significantly correlated with depression. About the type of treatment received up to the time of our survey, we found that patients who had received heavier treatment, particularly quadruple therapy, were more depressed than the others. Furthermore, most patients who had started their treatment more than a year earlier showed more depressive disorders than those who had started it less than a year. As for the psychological treatment associated with the medical-surgical treatment, the only two patients who benefited from it were depressed. However, this could be explained by the fact that this psychological treatment was initiated following the suspicion or confirmation of the diagnosis of depression in these patients (Table 2).

The last part of our study focused on the psychosocial and physical consequences after the discovery of breast cancer and their influence on the occurrence of depression. Psychological and physical discomforts presented by the fear of recurrence, the fear of death, the loss of autonomy, the alteration of body image, or the side effects of the treatment were significantly correlated with fewer depressive disorders. As for the socioeconomic and family repercussions, patients bothered by the alteration of their marital life, as well as by the loss of their family responsibilities, presented more depression. On the other hand, family carelessness did not seem to be a predictive factor for the occurrence of depressive disorders in our sample, as most of the women bothered by family carelessness were not depressed. All these results were statistically significant (*p* < 0.05) (Table 2).

**Figure 1:**
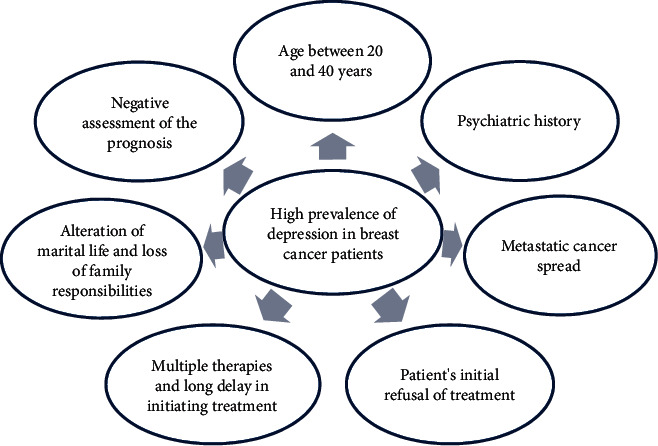
Representative diagram of the main factors associated with depression in breast cancer patients.

In light of what was demonstrated in the analytical study, we noted that the occurrence of the characterized depressive episodes significantly correlated with the following parameters: age between 20 and 40 years, the existence of psychiatric history, the metastatic spread of cancer, the patient's initial refusal of treatment, the patient's negative assessment of the prognosis, multiple therapy and a longer delay in initiating treatment, and the consequences of the repercussions on family life, in particular the alteration of marital life and the loss of responsibilities (Figure 1).

## 4. Discussion

Breast cancer is considered one of the neoplasms associated with a higher prevalence of depressive episodes. A meta-analysis by Reich et al. found a prevalence of major depressive episodes measured by DSM-IV criteria, ranging from 4.7% to 38% [10–12]. Another meta-analysis by Mitchell et al. found a prevalence of 14.1% [13].

At the national level, a few studies have been conducted on the psychological profile of cancer patients. To the best of our knowledge, no study specifically devoted to depressive disorders in breast cancer patients has been published to date. In 2010, a study concerning the evaluation of depressive disorders in 100 patients with cancer in different locations (including 31 breast cancers) found a prevalence of depression of 15% [14]. In addition, a study conducted over more than a year at the National Institute of Oncology in Rabat, looking at factors associated with psychological distress in a population of 446 Moroccan women suffering from breast cancer, found prevalence estimated at 26.9% [15]. In our study, among the 100 breast cancer patients interviewed, 26% had a characterized depressive episode diagnosed according to the MINI test. The severity of this episode, assessed by the BDI, was variable: mild in 42%, moderate in 50%, and severe depression in 2%. Thus, the prevalence rate found in our study was similar to that of the studies mentioned above.

Various factors affecting the occurrence of depressive disorders in breast cancer patients were identified. Data in the literature varied according to sample size, constructs evaluated, and populations examined. However, it remained difficult to identify a set of factors that were systematically linked to depression in this population [16].

Among the sociodemographic factors associated with an increase in psychological distress was a young age. Indeed, several studies have reported that younger breast cancer patients developed more anxiety and depressive disorders than older patients [17–19]. Ell et al. and Bardwell et al., studying depression in women with breast cancer for the first author and gynecological and breast cancers for the second, noted that women under 50 years of age were significantly more depressed than those over 50 [12, 16]. Our results were similar to those in the literature. Indeed, the majority of patients under 40 years of age were diagnosed with a major depressive episode. In contrast, no patient over 60 years of age was depressed. This difference was statistically significant (*p* < 0.05). However, other more recent studies have not found an association between the occurrence of depression and the age of cancer patients [13, 20].

Concerning marital status, some studies have found that women with breast cancer who were single, divorced, or widowed reported more psychological distress [21]. Indeed, Bardwell et al. found that married breast cancer patients were less depressed than those who were single [16]. This has been confirmed by other studies [22, 23]. The number of dependent children also appeared to be correlated with greater distress [24]. In our study, the majority of depressed patients were married and had one or more children. However, these two associations were not statistically significant. This could be explained by the predominance of married women (82%) with children (92% of them) in our sample.

A low level of education and a precarious professional status were sometimes identified as vulnerability factors [25]. On the other hand, for Akechi et al., a long education (9 years) was a significant factor associated with depression [26]. Studies by Ell et al. and Bardwell et al. found no significant correlation of depression with either education or occupation [12, 16]. Also in our study, no significant association could be found with these two parameters. Low socioeconomic status also seemed to be a predictive factor for psychological distress in women with breast cancer [25]. This was confirmed by several studies [26, 27], while other studies did not show a significant correlation [28]. Poor financial status could be a more important risk factor in case of lack of social security coverage. All of our patients had social security coverage, and 98% did not experience any discomfort with the cost of their medical care. Thus, we did not find a significant correlation of depression with socioeconomic status.

The existence of previous or additional health problems was also a risk factor for poorer psychological adaptation to this new cancer disease [24]. Indeed, Yun et al. demonstrated in their study of all cancers that comorbidities increased the risk of depression [29]. Ell et al. stated that the existence of joint comorbidity in breast cancer patients was significantly related to depression [12]. Contrary to these results, no significant association between the occurrence of depression and the presence or absence of medical and surgical history was observed in the patients of our study. On the other hand, the majority of our patients who claimed to have a psychiatric history had a characteristic depressive episode. This was statistically significant (*p* < 0.05). Several studies have highlighted the importance of psychiatric history, particularly depression, in terms of the risk of depressive relapse; this history should be systematically sought [30, 31]. Among these studies, Burgess et al. and Christensen et al. concluded that a personal history of anxiety or depressive disorders in women with breast cancer seemed to be predictive of psychological distress and depressive disorders during their cancer disease [17, 32]. Also, the family history of cancer was important, especially for breast cancer, where there was a genetic risk. The patient who has already gone through the bitter experience of this disease with her mother or sister will expect to go through the same suffering again [33]. In our study, women with a family history of breast cancer did experience this anxiety, but it was not significantly related to the occurrence of depressive disorders.

Announcing the diagnosis of breast cancer was a complex and rich task, on a case-by-case basis and in an interdisciplinary manner. In 2013, an American study showed that the announcement of a diagnosis of localized breast cancer resulted in 23% of symptoms leading to a “posttraumatic stress state”; most of these traumas occurred during the first consultation [34]. In our study, the situation that was reported most by the patients (89%) was the state of shock, followed by feelings of vulnerability or helplessness in 28% of the patients, then denial of the diagnosis (19%), fear of death (13%), feelings of guilt (3%), refusal of treatment (2%), and many other reactions. When analyzing these results, the patients who presented a state of shock had fewer depressive disorders; in contrast to those who refused treatment, they were diagnosed with the most severe depressive episode. Therefore, the announcement must be considered a real therapeutic act, as it was a key moment in the doctor-patient relationship that would profoundly determine the relationship of trust but also compliance with treatment, and the experience of the disease [35].

The risk of developing depression may also be related to the somatic manifestations of the cancer and its progression. Bardwell et al. found an impact of vasomotor and gastrointestinal disorders on depression in breast cancer patients [16]. Results from other studies indicated that fatigue, pain, and sleep disturbance were also frequently found to be predictive of depression or anxiety in this population [36–39]. In contrast, the study by Burgess et al. found no association between clinical symptoms of breast cancer and the development of anxiety or depressive disorders [16]. Similarly, our results were in line with those of Burgess et al.

As the cancer disease progressed and depended on the different stages of the treatment process, prevalence rates varied. Indeed, Burgess et al. estimated a prevalence of major depressive episodes of 33% at diagnosis, 24% at 3 months, and 15% one year after diagnosis [17]. Taking into account the stage of cancer, previous studies have estimated that about a quarter of all breast cancer patients had comorbid depression, with an estimate of 20-30% for early-stage cancer [40] and increasing rates of late-stage and palliative cancer (over 50%) [41]. Jacob et al. found a high risk of depression and anxiety in patients with metastatic cancer [42]. Another survey in the United States, which looked at anxiety episodes in young patients with metastatic breast cancer, estimated the prevalence to be 20% [43]. The results of our survey were consistent with those of the above studies [42, 43]. Indeed, patients with metastatic breast cancer were significantly more depressed (*p* < 0.05).

Psychosocial and family factors could also predict depression. Couples where the partners supported and showed understanding and affection towards their wives were better preserved and their sexual relationships may last longer [44]. On the other hand, some women were more concerned about their partners and/or children's experiences than about themselves [45], preferring to face this terrible deal alone. In 2012, a Moroccan study conducted at the National Institute of Oncology in Rabat, including 80 breast cancer patients, found that 68% of sexually active patients reported that cancer had changed their sex life. Various physical and psychological causes were reported, the most frequent being asthenia and lack of libido. In addition, 17% of the women had a change in marital status. However, none of the patients received a consultation with a psychosexologist or a psychiatrist [46]. In our survey, 19% of the patients reported an alteration in their marital life, and 32% suffered from sexual problems. The majority of women who were bothered by the alteration of their marital relationship were depressed. This association was statistically significant (*p* < 0.05). Thus, as we have seen, the occurrence of breast cancer disrupted not only the lives of patients but also those of their spouses. The impact of this cancer on the couple underlines the importance of considering both partners in this experience to support them, because, at the time of its occurrence, it was the couple that must face the disease and its consequences [47].

Furthermore, family support was known to play an important role in psychological and physical adjustment to the disease [48]. Bardwell et al., Wilson et al., and Nausheen and Kamal all found a significant correlation aversely related to depression: the more social support there was, the less depression was [16, 28, 49]. In this sense, a Moroccan study, including 446 patients followed for breast cancer, found that 26.9% of women were psychologically distressed. The lack of family support was the second factor independently associated with this distress [50]. In our study, the majority of patients bothered by their families' carelessness were not depressed. This association was nevertheless significant (*p* < 0.05). This can be explained by the predominance of mothers and married women in our sample who reported that their main causes of sociofamilial discomfort were mainly the alteration of marital life and difficulties in raising their children.

A study conducted in Norway objectified a prevalence of body image disorder of 30.6% in patients with breast cancer [51]. Another Tunisian study estimated that 45% of these patients suffered from body image disorder, which was also linked to impaired marital life and the occurrence of anxiety and depressive disorders [52]. According to Pikler and Winterowd, women who have a better self-image also have higher levels of confidence in coping with breast cancer [53]. As for our study, 64% of the patients in our sample reported discomfort with the alteration of body image. In contrast, the majority of these women were not depressed. The treatment modalities and the nature of the treatment were also risk factors for depression.

From breast surgery to chemotherapy to radiotherapy to hormonotherapy, the whole long process of care needed to be clearly explained [45]. The prospect of treatment is an important source of hope for the patient. Studies found that conservative surgery was more likely to result in depressive disorders [54, 55]. The increased prevalence and severity of depression in women undergoing chemotherapy have been documented in several studies [12, 25]. Similarly, Manzanera et al. found a prevalence of mood disorders of 55.6% in women treated with chemotherapy (plus or minus radiotherapy) compared to 19.4% in women treated with radiotherapy alone [56]. For patients undergoing hormone therapy, the study by Berglund et al. found more sexual difficulties than in women not undergoing this treatment [57] but without a significant risk of developing depression [58]. An encouraging finding was that of a study by Kissane et al. which found a prevalence of 9.6% in patients treated at an early stage of the disease and a prevalence of 6.5% in patients with advanced cancer [59]. This decrease in prevalence over the last two decades may reflect improvements in treatment, including less mutilating surgery, improved medical data, and a supportive social atmosphere [60]. In our study, the majority of patients who received heavier treatment, including quadruple therapy, as well as the majority who started treatment more than one year ago were depressed. This significant correlation (*p* < 0.05) can be explained by the importance of the physical and psychological suffering accumulated throughout the difficult treatment process, which sometimes exceeded ten years in some patients with metastatic cancer.

Several studies agreed that there is a link between psychological distress and psychiatric disorders and cancer prognosis [61–64]. Semimetal analyses, notably those of Chida et al. and Satin et al., agreed on the decreased survival in cancer patients with comorbid depression [62, 63]. In general, despite small but significant associations between depression and cancer prognosis, a causal relationship was again difficult to demonstrate due to the large number of overlapping symptoms between depression and cancer disease and the impact of treatment, both of which may be modulated by the underlying severity of cancer. In addition, a longitudinal study conducted over 11 years on 227 patients with breast cancer showed beneficial effects in terms of reduced risk of relapse and improved survival in patients who received long-term psychological care [64].

The risks were 1.5 to 2 times higher in women with breast cancer than in the general population. It was increased in the context of psychiatric disorders associated with cancer disease, particularly depression [24]. In a cohort from the Norwegian Cancer Registry between 1960 and 1999, 589 suicides were observed. The relative risk of suicide was double that of the general population and was highest in the first five months after the diagnosis. Most of these cancer patients committed suicide by overdosing on painkillers or sedatives prescribed by their doctors [65]. Other passive forms of suicide, which were frequent but remain ignored, should be highlighted, namely, the refusal of treatment and care [66]. Moreover, Farberow et al.'s study showed that 86% of suicides were committed during the preterminal and terminal phases of the disease [67]. Thus, the systematic search for factors aggravating depression, especially a personal or family history of attempted or completed suicide in any cancer patient was of paramount importance [24]. The clinician should be aware that talking about suicide with the patient would allow him or her to describe feelings, fears, and misconceptions and provide a sense of control [68].

Several hypotheses suggesting links between psychological factors and cancer have long been established [69]. However, the relationship between cancer disease and depression did not seem to be limited to psychosocial factors or those related to cancer treatment or progression. A 2016 study investigating the relationship between proinflammatory cytokines and major depression in cancer patients suggested the possibility that IL-1 may be a stronger predictor of “disease behaviours,” while IL-4 may be more specific to major depression [70]. Furthermore, it should be noted that the relationship between cancer and depression is reciprocal; in other words, it is depression, especially chronic depression, that may influence the occurrence or progression of cancer [71]. Studies are needed to better explain this association, particularly the “psychogenesis” of cancer. It is in this context that psychoneuroses-endocrine-immunology is involved and is opening up new horizons.

## 5. Conclusion

Despite the considerable number of studies conducted on this topic, determining the exact prevalence of depression in these breast cancer patients remains difficult. This is mainly due to differences in defining and measuring depression, patient populations, and cancer disease stages. In light of the results obtained, we can make a few suggestions aimed at improving the psychoontological management not only of women suffering from breast cancer but of all cancer patients, to prevent the occurrence of psychiatric pathologies that may compromise the ontological management and prognosis of the cancer disease in this population, such as the assignment of psychologists to oncology and radiotherapy departments, the programming of a psychiatric follow-up from the outset in the case of psychiatric history and at the slightest apparent suffering, and the training of medical staff and medical students in social psychology and medical communication, in particular concerning the announcement of a serious diagnosis.

The data obtained from our survey were limited due to the moderate size of the population studied and their profiles. Therefore, we hope this study will inspire other evaluations and comparative analyses of the prevalence of depression and its associated factors in breast cancer and other types of cancers at a national level. Such efforts could lead to more relevant results and a better understanding of the psychological suffering of cancer patients to improve their overall care.

## Figures and Tables

**Table 1:** Socioeconomic, clinical, and therapeutic characteristics of all participants.

Characteristics	Participants *n* = 100	
	*n*	%
Old (years)		
20-40	14	14
40-60	72	72
>60	14	14
Marital status		
Single	5	5
Married	82	82
Divorced	1	1
Widow	12	12
Education level		
Illiterate	45	45
Primary	29	29
Secondary	19	19
Superior	7	7
Professional status		
Employed	13	13
Retired	5	5
Unemployed	82	82
Area of living		
Urban	93	93
Rural	7	7
Socioeconomic level		
Low	39	39
Medium	57	57
High	4	4
With or without children		
With	92	92
Without	8	8
Personal history		
Medical history	25	25
Surgical history	41	41
Psychiatric history	15	15
Family history		
Breast cancer	17	17
Psychiatric history	12	12
Location of cancer		
Unilateral	94	94
Bilateral	6	6
Stage of cancer dissemination		
Local	25	25
Locoregional	51	51
Metastatic	24	24
Circumstances of discovery		
Self-examination	89	89
Fortuitous discovery	8	8
Control of benign mastopathy	1	1
Screening campaign	2	2
Type of treatment received		
Chemotherapy only	18	18
Surgery+chemotherapy	28	28
Surgery+radiotherapy	11	11
Surgery+chemotherapy+radiotherapy	20	20
Surgery+chemotherapy+hormonotherapy	5	5
Surgery+chemotherapy+radiotherapy+hormonotherapy	18	18
Care and psychological support		
Yes	2	2
No	98	98
Depressive episode prevalence based on the MINI test		
Yes	26	26
No	74	74
Depressive episode intensity based on the BDI (*n* = 26)		
Mild	11	11
Moderate	13	13
Severe	2	2

Qualitative variables were expressed as headcount (*n*) and percentage (%).

**Table 2:** Main significant analytical results of the depression prevalence according to sociodemographic, clinical, and therapeutic characteristics of breast cancer patients.

Sample characteristics	Depression		No depression		*p* value (*p*)
	*n* = 26	%	*n* = 74	%	
Old (years)					
20 to 40	8	57.1	6	42.9	**0.004**
41 to 60	18	25	54	75	0.715
>60	0	0	14	100	**0.017**
Psychiatric history					
Yes	11	64.7	6	35.3	**0.000**
No	15	18.1	68	81.9	
Stage (dissemination)					
Local	2	8	23	92	**0.018**
Locoregional	11	21.6	40	78.4	0.303
Metastasis	13	54.2	11	45.8	**0.000**
Treatment refusal					
Yes	2	100	0	0	**0.016**
No	24	24.5	74	75.5	
Prognosis assessment by the patient					
Curable	22	22.9	74	77.1	**0.001**
Not curable	4	100	0	0	
Alteration of marital life					
Yes	11	55	9	45	**0.001**
No	15	18.8	65	81.2	
Loss of family responsibilities					
Yes	11	52.4	10	47.6	**0.002**
No	15	29	64	81	
Kind of treatment					
Chemotherapy only	4	22.2	14	77.8	0.687
Surgery and chemotherapy	5	17.9	23	82.1	0.247
Surgery and radiotherapy	1	9.1	10	90.9	0.175
Surgery, chemotherapy, and radiotherapy	2	10	18	90	0.068
Surgery, chemotherapy, and hormonotherapy	4	80	1	20	**0.005**
Surgery, chemotherapy, radiotherapy, and hormonotherapy	10	55.6	8	44.4	**0.002**
Time to start treatment					
<3 months	3	9.1	30	90.9	**0.007**
Between 3 months and 1 year	9	20.9	34	79.1	0.315
Between 1 year and 5 years	7	58.3	5	41.7	**0.006**
>5 years	7	58.3	5	41.7	**0.006**
Psychological care					
Yes	2	100	0	0	**0.016**
No	24	24.5	74	75.5	**0.007**

Qualitative variables were expressed as headcount (*n*) and percentage (%); the bolded value means a significant *p* value.

## Data Availability

The data used to support the findings of this study are included within the article.

## Abbreviations

IDB: Beck Depression Inventory
MINI: Mini International Neuropsychiatric Interview
WHO: World Health Organization.
